# Functional connectivity-based subtypes of individuals with and without autism spectrum disorder

**DOI:** 10.1162/netn_a_00067

**Published:** 2019-02-01

**Authors:** Amanda K. Easson, Zainab Fatima, Anthony R. McIntosh

**Affiliations:** Rotman Research Institute, Baycrest Hospital, Toronto, ON, Canada; Department of Psychology, University of Toronto, Toronto, ON, Canada; Department of Psychology, Faculty of Health, Sherman Health Sciences Centre, York University, Toronto, ON, Canada; Rotman Research Institute, Baycrest Hospital, Toronto, ON, Canada; Department of Psychology, University of Toronto, Toronto, ON, Canada

**Keywords:** Autism spectrum disorder, Functional connectivity, Clustering, Brain-behavior relationships, Multivariate statistics, Resting-state networks

## Abstract

Autism spectrum disorder (ASD) is a heterogeneous neurodevelopmental disorder, characterized by impairments in social communication and restricted, repetitive behaviors. Neuroimaging studies have shown complex patterns and functional connectivity (FC) in ASD, with no clear consensus on brain-behavior relationships or shared patterns of FC with typically developing controls. Here, we used a dimensional approach to characterize two distinct clusters of FC patterns across both ASD participants and controls using *k*-means clustering. Using multivariate statistical analyses, a categorical approach was taken to characterize differences in FC between subtypes and between diagnostic groups. One subtype was defined by increased FC within resting-state networks and decreased FC across networks compared with the other subtype. A separate FC pattern distinguished ASD from controls, particularly within default mode, cingulo-opercular, sensorimotor, and occipital networks. There was no significant interaction between subtypes and diagnostic groups. Finally, a dimensional analysis of FC patterns with behavioral measures of IQ, social responsiveness, and ASD severity showed unique brain-behavior relations in each subtype and a continuum of brain-behavior relations from ASD to controls within one subtype. These results demonstrate that distinct clusters of FC patterns exist across ASD and controls, and that FC subtypes can reveal unique information about brain-behavior relationships.

## Author Summary

Autism spectrum disorder (ASD) is a neurodevelopmental disorder, with high variation in the types of severity of impairments in social communication and restricted, repetitive behaviors. Neuroimaging studies have shown complex patterns of communication between brain regions, or functional connectivity (FC), in ASD. Here, we defined two distinct FC patterns and relationships between FC and behavior in a group of participants consisting of individuals with and without ASD. One subtype was defined by increased FC within distinct networks of brain regions and decreased FC between networks compared with the other subtype. A separate FC pattern distinguished ASD from controls. The interaction between subtypes and diagnostic groups was not significant. Dimensional analyses of FC patterns with behavioral measures revealed unique information about brain-behavior relations in each subtype.

## INTRODUCTION

Autism spectrum disorder (ASD) is a neurodevelopmental disorder that is characterized by impairments in social cognition as well as restricted and repetitive behaviors (RRBs; American Psychiatric Association, 2013). ASD is highly heterogeneous, with a broad range of the types and severities of behaviors that can be displayed. For instance, verbal and nonverbal IQ are highly variable in ASD (e.g., Munson et al., 2008), and RRBs can range from low-level stereotyped motor behaviors to higher order behaviors such as insistence on sameness (American Psychiatric Association, 2013). It has been proposed that these complex behavioral features are associated with atypical patterns of functional connectivity (FC). Such theories include reduced communication between frontal and posterior brain regions (Just et al., 2012), increased local FC along with reduced long-range FC (Belmonte et al., 2004; Courchesne & Pierce, 2005), and an abnormal developmental trajectory of FC compared with typically developing (TD) individuals (Nomi & Uddin, 2015; Uddin et al., 2013b). However, complex patterns of both increased and decreased FC have been found in neuroimaging studies of ASD, and results are inconsistent across studies (see Hull et al., 2016, Picci et al., 2016, and Uddin et al., 2013b, for reviews).

It is crucial to consider the heterogeneous nature of ASD, both in terms of behavioral severity and FC profiles. The importance of this consideration is highlighted by the inconsistent results regarding relationships between FC and behavioral profiles in individuals with ASD in previous studies (e.g., Keown et al., 2013; Lee et al., 2016; Monk et al., 2009; Uddin et al., 2013b). Several recent studies that considered the heterogeneity of neurobiological and behavioral features of ASD have reported novel findings regarding brain-behavior relationships. Hahamy, Behrmann, and Malach (2015) found that idiosyncratic distortions in FC from a “typical” template were related to ASD symptom severity. Nunes et al. (2018) reported that incorporation of vertices along the cortical surface into intrinsic connectivity networks, particularly into default mode and sensorimotor networks, was more idiosyncratic in ASD and related to symptom severity.

FC-based subtypes have the potential to resolve some of the current discrepancies regarding the nature of FC abnormalities in individuals with ASD, and to shed light on the complex relationships between FC and behavior, which may differ between subtypes. Previously, ASD subtypes have been defined based on clusters of social communication behaviors and RRBs (Georgiades et al., 2013), structural MRI (Hrdlicka et al., 2005), various neuroanatomical features (Hong et al., 2017), and FC (Chen et al., 2015). Chen et al. (2015) found two subtypes that exhibited unique FC patterns in different resting-state networks (RSNs), and differed in terms of ASD symptom severity. Hong et al. (2017) found that prediction of individual scores on the Autism Diagnostic Observation Schedule (ADOS) greatly improved when subtypes were considered, compared with considering all ASD participants as one group. Thus, brain-based subtyping has the potential to elucidate brain-behavior relationships that are unique to each subtype, as certain behaviors may result from complex interplay between local and distributed processing in the brain. One limitation of these studies is that they did not include both ASD and TD participants in the subtyping procedures. Because of the heterogeneity of ASD symptomatology and inconsistent reports of FC profiles in ASD, it is important to consider FC patterns that may be shared among those with ASD and controls. Recent work revealed shared FC patterns between ASD and TD participants, and between TD individuals and other clinical groups, including ADHD and schizophrenia (Spronk et al., 2018). Furthermore, taking a dimensional approach to examining FC can reveal information about brain-behavior relationships that exist as a continuum across typical development and clinical diagnoses. Rashid et al. (2018) demonstrated a continuum of the relationship between neurobiological features and subclinical ASD symptoms in healthy controls. Additionally, Muller and Amaral (2017) highlighted the importance of “studying functional systems dimensionally within the [research domain criteria] framework” and defining biological subtypes of ASD to make progress toward customized treatments and behavioral interventions.

In the present study, we used a data-driven, dimensional approach to characterize subtypes based on distinct clusters of FC in all participants, and to relate FC patterns to specific behavioral profiles in these subtypes. We used *k*-means clustering, an unsupervised machine learning technique, to define subtypes by using functional connections as features. Next, we implemented multivariate statistical analyses that, when applied to neuroimaging data, reveal optimal relationships between measures of brain activity and experimental design or group membership. Using this approach categorically, we characterized connections that were reliably different between subtypes, and between ASD and TD participants. We also characterized dimensional relationships between particular FC patterns and a set of behaviors across participants in both diagnostic groups within each subtype. It was hypothesized that defining FC-based subtypes in a sample of both ASD and TD participants by using data-driven metrics would reveal unique information about brain-behavior interactions.

## RESULTS

### FC-Based Subtypes of ASD and TD Participants

FC-based subtypes were defined using *k*-means clustering. It was necessary to regress the effects of age and acquisition site out of the FC matrices prior to performing *k*-means clustering (see Materials and Methods). When these effects were not removed, there was a significant difference in the distribution of scan sites between the two subtypes defined by *k*-means clustering, *X*^2^ (4, *N* = 266) = 78.60, *p* < 0.001. After the scan sites were regressed from the data, the resulting subtypes were significantly different in age, *t*(264) = 2.50, *p* = 0.01; thus, effects of both site and age were regressed from the data.

**Figure 1.**
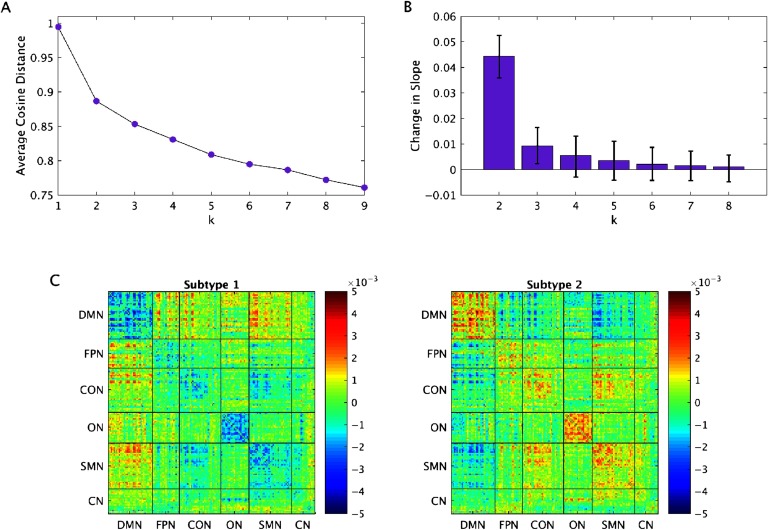
(A) Elbow point plots, indicating that the optimal number of clusters is 2. (B) Bootstrapping procedure to evaluate the reliability of the optimal number of clusters. Error bars show 95% confidence intervals. (C) Subtype centroids. DMN = default mode network; FPN = fronto-parietal network; CON = cingulo-opercular network; ON = occipital network; SMN = sensorimotor network; CN = cerebellar network.

The optimal number of clusters, as determined by the elbow point criterion, was 2 (Figure 1A). Using a bootstrapping procedure to evaluate the reliability of the optimal number of clusters, it was found that the optimal number of clusters was 2 in 500/500 bootstrap samples (Figure 1B). Qualitatively, it can be seen that the change in slope for *k* = 2 was always much greater than the change in slope for any other value of *k*. For example, the mean change in slope for *k* = 2 was 4.81 times greater than that for *k* = 3, and confidence intervals do not overlap. For values of *k* = 3 or more, the intervals do overlap.

Subtype 1 consisted of 85 ASD participants and 54 TD participants. Subtype 2 consisted of 60 ASD participants and 67 TD participants. Qualitatively, Subtype 1 was defined by stronger FC between networks, particularly between the default mode network (DMN) and other networks, and weaker FC within networks relative to Subtype 2 (Figure 1C).

Importantly, subtypes did not differ in demographics or behavior, including IQ, eye status, medication use, presence of comorbidities, head motion, or the parameters (scan site and age) that were regressed out of the FC matrices (Supporting Information Table S3, Easson, Fatima, & McIntosh, 2019). Although subtypes differed in ADOS communication scores (*t*(112) = 2.62, *p* = 0.01), they were marginally significantly different in ADOS total scores (*t*(130) = 1.87, *p* = 0.06) and differences in the social responsiveness scale (SRS; Constantino & Gruber, 2005) scores approached significance (*t*(136) − 1.71, *p* = 0.09), these differences were driven by the fact that there were more TD participants with these scores in Subtype 2 compared with Subtype 1. SRS scores did not differ between ASD participants in Subtypes 1 and 2, and also did not differ between TD participants in Subtypes 1 and 2. ADOS scores did not differ between ASD participants in Subtypes 1 and 2, but could not be compared for TD participants in Subtypes 1 and 2 because ADOS scores were only available for 2 TD participants in Subtype 1 and 12 TD participants in Subtype 2.

### Multivariate Analysis of Subtype and Diagnostic Group Differences in FC

**Figure 2.**
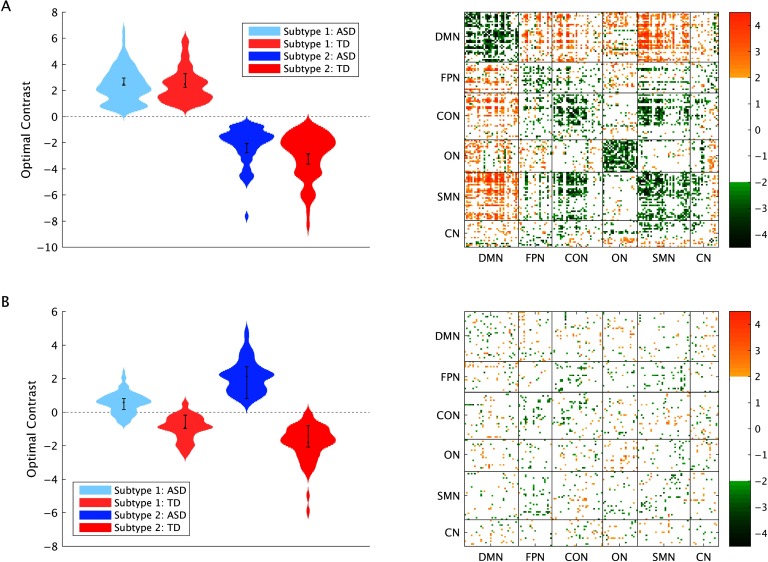
Results from the multivariate group analysis. (A) First pattern and (B) second pattern, and the associated BSRs for each connection at a threshold of ±2. Error bars show 95% confidence intervals determined through bootstrap resampling.

We used a multivariate statistical approach to determine differences in FC between subtypes and between ASD and TD participants. The reliability of these patterns was determined via bootstrap sampling. A functional connection was considered to be reliable, or stable, if the absolute value of its bootstrap ratio (BSR) exceeded 2. This analysis revealed two signifcant patterns.The first pattern showed stable differences in FC between subtypes (*p* < 0.001, 61.07% of variance explained, Figure 2A), whereby Subtype 2 was characterized by stronger FC within RSNs, and weaker FC between RSNs, compared with Subtype 1. The contrast expression for this FC pattern (Supporting Information Figure S3, Easson et al., 2019) revealed that functional connections with significant positive BSRs, on average, were positive in Subtype 1 and negative in Subtype 2, and vice versa for negative BSRs. The second pattern revealed a contrast between diagnostic groups in both subtypes (*p* = 0.02, 21.74% of variance explained, Figure 2B), with a diffuse spatial pattern. The contrast expression (Supporting Information Figure S4, Easson et al., 2019) revealed that functional connections with significant positive BSRs, on average, were negative in the ASD group and positive in the TD group, and vice versa for negative BSRs. The third pattern, which revealed a subtype by diagnosis interaction, was not significant, *p* = 0.92.

**Figure 3.**
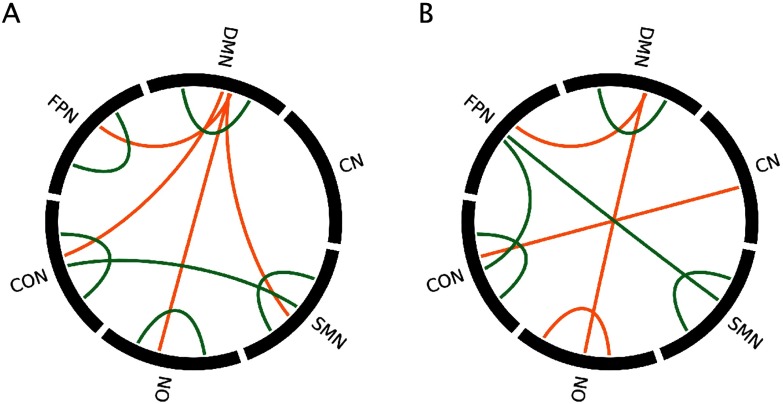
Significant contributions of RSN pairs to each pattern for positive and negative BSRs, for the (A) first pattern and (B) second pattern from the multivariate group analysis. Orange = positive BSRs, green = negative BSRs.

In addition to examining individual connections that differed between subtypes (first spatial pattern) and diagnostic groups (second spatial pattern), the significance of the average spatial patterns within and between RSNs was evaluated using permutation tests (see Materials and Methods) and is shown in Figure 3.

### Multivariate Analyses of FC-Behavior Relationships

A multivariate brain-behavior analysis was used to assess relationships between FC and a set of behavioral measures in the two subtypes, including IQ, ADOS scores (communication; COMM), social affect, and RRB, and scores on the SRS. The full set of behavioral measures was available for 51 participants (49 ASD, 2 TD) in Subtype 1 and 50 participants (38 ASD, 12 TD) in Subtype 2. ADI-R scores were not included, as only 28 participants in Subtype 1 and 26 participants in Subtype 2 had the full set of behavioral measures including ADI-R scores. Furthermore, none of the participants with the full set of scores including ADI-R scores were TD participants.

The analysis revealed three significant patterns. The first pattern (*p* = 0.03, 32.09% covariance explained) revealed stable relationships between FC and IQ and ADOS RRB scores in Subtype 1, and stable relationships between FC and all behavioral measures in Subtype 2. This first pattern was a contrast between Subtypes 1 and 2 in terms of relationships with FC and ADOS RRB scores, such that connections that were reliably positively correlated with ADOS RRB scores in Subtype 1 were negatively correlated in Subtype 2, and vice versa. The next significant pattern was the third pattern (*p* = 0.008, 10.82% covariance explained), which revealed a different spatial pattern that exhibited stable correlations with IQ and SRS in Subtype 1, and with all ADOS scores and SRS in Subtype 2. Additionally, there was a contrast between Subtypes 1 and 2 in terms of correlations between FC and SRS scores. The seventh pattern (*p* = 0.003, 4.45% covariance explained) revealed a contrast between Subtypes 1 and 2 in terms of correlations between FC and ADOS communication scores, as well as stable correlations between FC and ADOS social affect scores in Subtype 1.

**Figure 4.**
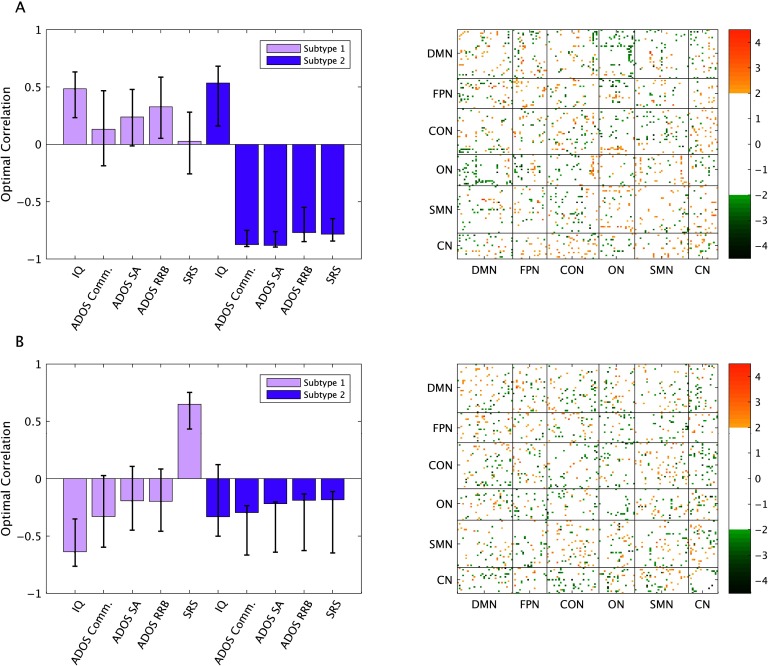
Results from the multivariate brain-behavior analysis. (A) First pattern and (B) third pattern, and the associated BSRs for each connection at a threshold of ±2. Error bars show 95% confidence intervals determined through bootstrap resampling.

**Figure 5.**
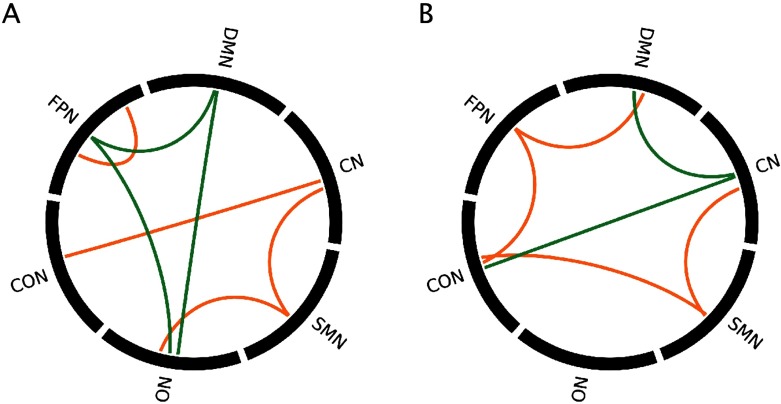
Significant contributions of RSN pairs to each pattern for positive and negative BSRs for 9A) first pattern and (B) third pattern. Orange = positive BSRs, green = negative BSRs.

Overall, it can be seen that connections that show stable correlations with behavior are diffuse. Patterns that accounted for more than 10% of the covariance between FC and behavior (i.e., patterns 1 and 3) are shown in Figure 4, and the corresponding contrast expressions are shown in Supporting Information Figure S5 and S6 (Easson et al., 2019). The stability of these FC-behavior relationships within and between RSNs are shown in Figure 5.

### Continuum of FC-Behavior Relationships Across Diagnostic Groups

**Figure 6.**
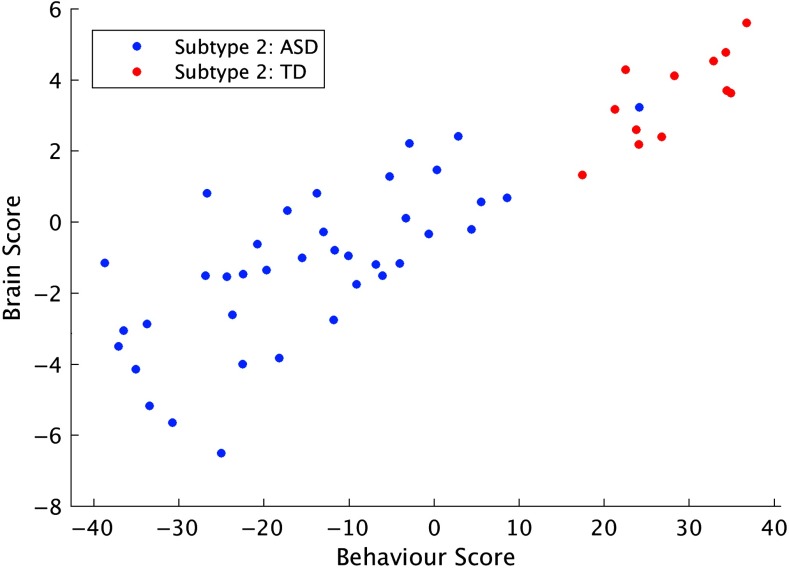
Brain and behavior scores for Subtype 2, from the first pattern of the multivariate brain-behavior analysis.

The relationship between brain and behavior scores for ASD and TD participants in Subtype 2 was evaluated for the first pattern of the multivariate brain-behavior analysis, which explained the greatest proportion of covariance between FC and the set of behavioral measures. The continuum of scores for both brain and behavior variables illustrates that there is a pattern of FC that covaries with the severity of behaviors across the autism spectrum and typical development (Figure 6). This analysis was only performed in Subtype 2, as there were only 2 TD participants in Subtype 1 who had the full set of behavior measures.

### Relationships Between Multivariate Group and Brain-Behavior Spatial Patterns

We then determined the relationship between the patterns from the multivariate group analysis and the multivariate brain-behavior analysis by correlating the brain saliences for each analysis, and evaluated the significance of these correlations using permutation testing. There was a significant correlation between the first brain-behavior pattern and the second group pattern (*r* = 0.40, *p* < 0.001), indicating that the continuum of FC-behavior relationships was associated with the diagnostic pattern from the group analysis. The correlations between the other patterns were not significant (brain-behavior pattern 1 and group pattern 1: *r* = −0.06, *p* = 0.81; brain-behavior pattern 3 and group pattern 1: *r* = 0.005, *p* = 0.45; brain-behavior pattern 3 and group pattern 2: *r* = 0.07, *p* = 0.13.

## DISCUSSION

### Overview

This study reveals distinct clusters of FC patterns across a cohort of both ASD and TD participants. We characterized differences in FC between subtypes and diagnostic groups, and showed that individuals within each subtype exhibit different relationships between FC and behavior. The continuum of brain and behavior scores across ASD and TD participants reveals that FC phenotypes observed in ASD extend to typical development in relation to behavioral severity.

### Comparison of FC Between Subtypes and Diagnostic Groups

Two subtypes were defined for all participants. When all four groups were considered in a multivariate analysis (i.e., ASD Subtype 1, ASD Subtype 2, TD Subtype 1, and TD Subtype 2), the strongest pattern, not surprisingly, was a contrast between subtypes. Regardless of diagnostic group, Subtype 2 was defined by greater FC within networks and lower FC between networks, especially between the DMN and other RSNs, compared with Subtype 1. Connections within networks tended to be positive on average in Subtype 2 and negative in Subtype 1, indicating reduced interactions among brain regions within these networks in Subtype 1. Connections between networks that were lower in Subtype 2 tended to be negative, but were positive on average in Subtype 1 (Supporting Information Figure S3, Easson et al., 2019). As anticorrelations between RSNs are hypothesized to signify the ability for regions that are relevant for certain cognitive functions to become activated with concurrent deactivation of irrelevant regions (Fox et al., 2005; Greicius et al., 2003), these abilities may be affected in Subtype 1. Using the Power atlas (Power et al., 2011), previous FC-based ASD subtypes also revealed unique FC profiles between subtypes within and between RSNs (Chen et al., 2015).

A second pattern revealed diffuse functional connections that differed between diagnostic groups in both subtypes. ASD participants showed decreased FC within the sensorimotor network (SMN), DMN, and cingulo-opercular network (CON), but greater FC within the occipital network (ON). Atypical FC of sensorimotor regions has been reported in ASD (Anderson et al., 2011a; Mostofsky et al., 2009; Turner et al., 2006). Abnormal DMN functioning in ASD has been related to difficulties with self-referential processing, redirecting attention from external to internal processing, and theory of mind (e.g., Assaf et al., 2010). Various studies have reported decreased FC between DMN regions in ASD (Assaf et al., 2010; Kennedy & Courchesne, 2008; Monk et al., 2009; Weng et al., 2010), although hyperconnectivity has also been reported (Monk et al., 2009; Uddin et al., 2013a). Decreased FC within the CON is in line with previous studies that showed difficulties with set maintenance in ASD (Kaland, Smith, & Mortensen, 2008; Miller et al., 2015). Increased FC in the ON is consistent with findings of increased local connectivity in primary visual regions (Keown et al., 2013) and increased involvement of extrastriate cortex (Shen et al., 2012) in ASD. Additionally, increased FC was found between the DMN and FPN, DMN and ON, and CON and CN in ASD participants. These connections were positive on average in ASD, but negative on average in controls (Supporting Information Figure S4, Easson et al., 2019). Previous studies have reported reduced negative connectivity in ASD, which was described as reduced functional segregation of networks (Rudie et al., 2012, 2013a). However, other between-network connections (FPN-CON and FPN-SMN) exhibited a greater degree of anti-correlation in ASD. The functional significance of decreased anticorrelations between some RSNs, but increased anticorrelations between others, remains to be explored.

The third pattern, showing a subtype by diagnosis interaction, was not significant, thus revealing additive effects of subtype and diagnosis on FC patterns. Therefore, the expression of the subtypes does not depend on the diagnosis; the manifestation of the subtypes in ASD is not different from controls.

### Comparison of FC-Behavior Relationships Between Subtypes

Reliable correlations between FC and behavior were observed within and between RSNs for IQ and ADOS RRB scores for Subtype 1, and all behavioral measures for Subtype 2, showing that similar behavioral profiles can be associated with different functional correlates in the brain. Previous studies have reported mixed results regarding FC-behavior relationships in ASD (e.g., Keown et al., 2013; Lee et al., 2016; Monk et al., 2009; Uddin et al., 2013b). For instance, Lee et al. (2016) reported a negative correlation between RRBs and connections involving certain DMN regions, whereas Monk et al. (2009) reported a positive correlation. Our results highlight the importance of considering FC-based subtypes when examining brain-behavior relationships in ASD and controls. Importantly, individuals in each subtype did not differ significantly in IQ or SRS scores, and ASD participants in the two subtypes did not differ significantly in ADOS scores. Thus, there is unique information about FC-based subtypes that is not accessible by using behavior alone. This finding of unique brain-behavior relationships in different subtypes is supported by previous work that showed that prediction of ADOS scores improved when subtypes of ASD, based on unique neuroanatomical profiles, were considered, as opposed to considering all ASD participants as a single group (Hong et al., 2017). This study, as well as the FC-based subtypes study by Chen et al. (2015), also utilized data from the Autism Brain Imaging Data Exchange (ABIDE); thus, it is possible that there is overlap in the participants in our study and these previous studies.

The multivariate brain-behavior analysis supports the idea that instead of being a categorical diagnosis, ASD should indeed be considered as an extreme of a continuum of both neurobiological and behavioral features that can also be observed in TD individuals (Constantino & Todd, 2003; Rashid et al., 2018). In other words, there is normal variation in FC across both ASD and TD participants (Figure 6), but too much of this natural variation is associated with a diagnosis of ASD. This idea is supported by the continuum of brain and behavior scores from pattern 1 of the brain-behavior analysis for Subtype 2, and the significant correlation between the spatial pattern for this pattern and the second pattern from the group analysis, that is, the contrast in FC between diagnostic groups.

This dimensional approach has also been reinforced by ASD studies that accounted for heterogeneity of the relationships between behavior and various neurobiological features (Hahamy et al., 2015; Nunes et al., 2018). Recently, it has been noted that different features of brain function are variable even among TD individuals, and a certain feature cannot be considered to be an impairment unless it is accompanied by behavioral symptoms (Muller & Amaral, 2017). Our results support this idea by showing that some FC patterns are (a) similar among subsets of ASD and TD participants and (b) correlated with behavioral severity. The similarity of FC patterns in ASD and controls has also been demonstrated by Spronk et al. (2018), who showed that FC patterns between TD participants and several clinical groups, including ASD, ADHD, and schizophrenia, are highly correlated.

### Limitations

One limitation of our study is that we defined subtypes using a single data preprocessing strategy. However, inconsistent results between FC studies in ASD relate, in part, to different preprocessing choices (Hull et al., 2016). For instance, Ciric et al. (2017) showed that global signal regression reduces the relationship between FC and head motion, but can result in distance-dependent artifacts in FC unless used in combination with censoring methods. Global signal regression and low-pass filtering have been shown to affect group differences in FC between participants with and without ASD (Gotts et al., 2013; Muller et al., 2011). The length of fMRI scans may also contribute to heterogeneity across studies: increasing scan lengths improves the reliability of FC estimates (Birn et al., 2013). It is therefore crucial to gain a better understanding of how preprocessing choices and scanning parameters affect group differences in FC, and to compare FC-based subtypes across different preprocessing strategies.

Unfortunately, with the current dataset, we did not have a way to investigate age, as when age was not controlled for in the FC data, the resulting subtypes from *k*-means clustering differed significantly in age. Therefore, we cannot rule out the possibility that FC-based subtypes may manifest differently in various age groups. Uddin et al. (2013b) reported complex developmental trajectories of FC in ASD, with primarily hyperconnectivity in childhood and hypoconnectivity in adulthood. Furthermore, Anderson et al. (2011b) found that classification accuracy of ASD based on FC was higher in younger compared with older cohorts, suggesting that FC patterns between ASD and controls may be more distinct in childhood. Thus, although unique FC subtypes exist across a broad age range, there may be differences in FC clusters in children, adolescents, and adults. In this study, age ranges differed between scan sites, making it difficult to differentiate between age and site effects on FC. Larger samples within smaller age ranges from a single site are therefore required to study the manifestation of subtypes in different age groups.

Furthermore, we examined the continuum of brain and behavior scores across both ASD and TD participants in Subtype 2; however, ADOS scores were available for only 2 TD participants in Subtype 1. To better characterize the continuum of FC-behavior relationships across participants in different subtypes, future studies should collect measures of subclinical ASD behaviors using scales such as the SRS and Autism-Spectrum Quotient (AQ; Baron-Cohen et al., 2001), which is a brief self-report of ASD traits.

Finally, ABIDE consists of data from high-functioning individuals with ASD, thus limiting the generalizability of our findings. Including individuals with low-functioning ASD will be important in future studies to determine if similar subtypes exist among these individuals.

### Conclusions

Multivariate analyses of FC-based subtypes highlight the importance of considering the heterogeneity of FC patterns and behavior, and reveal the continuum of brain-behavior relationships in individuals with and without ASD. As subtypes exhibited different relationships between FC and behavior, it will be important to determine if individuals with ASD in different subtypes exhibit unique responses to treatments and behavioral therapies.

## MATERIALS AND METHODS

### Participants

Resting-state fMRI data from 145 males with ASD and 121 TD males were acquired from the Preprocessed Connectomes Project (Craddock et al., 2013; http://www.preprocessed-connectomes-project.org/abide). The data had been obtained from ABIDE (Di Martino et al., 2014; http://www.fcon_1000.projects.nitrc.org/indi/abide) and preprocessed using the Connectome Computation System (CCS) pipeline (Xu et al., 2015). Participants were excluded if their age was greater than 40, full scale IQ was less than 75, mean framewise displacement (FD) during the resting-state fMRI scan was greater than 0.20 mm, percentage of data points exceeding 0.20 mm was greater than 20%, and/or scans were rated as good by less than two (out of 3) raters as per the ABIDE quality assessment protocol (http://preprocessed-connectomes-project.org/abide/quality_assessment.html). Groups were matched for age, IQ, mean FD, and the percentage of data points exceeding 0.20 mm. ASD diagnoses were confirmed using ADOS (Lord et al., 2000) and/or the Autism Diagnostic Interview-Revised (ADI-R; Lord et al., 1994). Participant characteristics are shown in Table 1, along with the number of scores that were available for ADOS, ADI-R, and SRS if these scores were not available for all participants. Participant characteristics for each site are described in Supporting Information Table S1 (Easson et al., 2019).

**Table 1.** Participant characteristics.

**Variable**	**ASD Mean ± *SD* [range]**	**TD Mean ± *SD* [range]**	**Significance**
*N*	145	121	
Age	16.47 ± 6.46 [7.13–39.10]	16.03 ± 5.70 [6.47–31.78]	*t*(264) = 0.58, *p* = 0.56
IQ	107.57 ± 16.32 [76–148]	110.08 ± 11.61 [80–133]	*t*(264) = −1.43, *p* = 0.15
Mean FD	0.07 ± 0.04 [0.02–0.19]	0.07 ± 0.03 [0.03–0.19]	*t*(264) = 1.32, *p* = 0.19
Percent FD > 0.2 mm	4.69 ± 5.27 [0–19.33]	3.92 ± 1.29 [0–19.33]	*t*(264) = 1.29, *p* = 0.20
Handedness	120 RH 21 LH	109 RH 10 LH	*X*^2^(1, *N* = 266) = 0.52, *p* = 0.13
Eye status	121 open 24 closed	95 open 26 closed	*X*^2^(1, *N* = 266) = 1.38, *p* = 0.35
Scan site	NYU: 59 SDSU: 11 TRINITY: 18 UM: 26 USM: 31	NYU: 52 SDSU: 10 TRINITY: 16 UM: 29 USM: 14	*X*^2^(4, *N* = 266) = 5.07, *p* = 0.28
Medication use	27 yes 86 no 32 unknown	0 yes 106 no 15 unknown	N/A
Comorbidities	28 yes 117 no/unknown	0 yes 121 no/unknown	N/A
ADOS Total	11.69 ± 3.68 [5–22] (*N* = 118)	1.14 ± 1.17 [0–4] (*N* = 14)	*t*(130) = 10.64, *p* < 0.001
ADOS Communication	3.89 ± 1.55 [0–8] (*N* = 100)	0.50 ± 0.65 [0–2] (*N* = 14)	*t*(112) = 8.06, *p* < 0.001
ADOS Social	7.89 ± 2.81 [2–14] (*N* = 100)	0.64 ± 0.84 [0–3] (*N* = 14)	*t*(112) = 9.56, *p* < 0.001
ADOS RRB	2.04 ± 1.46 [0–7] (*N* = 98)	0.07 ± 0.27 [0–1] (*N* = 14)	*t*(110) = 5.00, *p* < 0.001
ADI-R Social	19.07 ± 5.44 [7–30] (*N* = 108)	N/A	N/A
ADI-R Verbal	15.38 ± 4.36 [2–25] (*N* = 109)	N/A	N/A
ADI-R RRB	5.66 ± 2.60 [0–12] (*N* = 109)	N/A	N/A
SRS	92.56 ± 31.00 [26–164] (*N* = 89)	20.59 ± 12.43 [1–56] (*N* = 49)	*t*(136) = 15.56, *p* < 0.001

### fMRI Preprocessing

Data from five sites (New York University Lagone Medical Center, University of Utah School of Medicine, San Diego State University, Trinity Centre for Health Sciences, and University of Michigan) using a TR of 2,000 ms were included. The proportion of ASD compared with TD subjects was not significantly different across sites, *X*^2^(4, *N* = 266) = 5.07, *p* = 0.28. Written, informed consent or assent was obtained for all participants in accordance with respective institutional review boards. Additional information about scanner types and parameters can be found on the ABIDE website (http://www.fcon_1000.projects.nitrc.org/indi/abide). The CCS preprocessing steps, which had been carried out as part of the Preprocessed Connectomes Project, were as follows: dropping the first four volumes, removing and interpolating temporal spikes, slice timing correction, motion correction, brain mask creation, 4D global mean-based intensity normalization, boundary-based registration of functional to anatomical images, anatomical segmentation of gray matter, white matter and cerebrospinal fluid, nuisance parameter regression (including 24 motion parameters, white matter and CSF signals, linear and quadratic trends, and the global signal), band-pass filtering (0.01 to 0.1 Hz), and registering functional images to the MNI template. The final preprocessed time series for each subject were obtained from the Preprocessed Connectomes Project. We chose to use data that had the global signal regressed out, as this step has been shown to help mitigate differences across multiple sites (Power et al., 2014). Furthermore, it has been shown recently that global signal regression attenuates artifactual changes in BOLD signal that are introduced by head motion (Byrge & Kennedy, 2017; Ciric et al., 2017; Power et al., 2017). It should also be noted that without global signal regression, FC-based subtypes differed in head motion (both mean FD, *t*(264) = −4.68, *p* < 0.001, and percentage of frames above 0.2 mm, *t*(264) = −5.02, *p* < 0.001). We also implemented ICA denoizing by using ICA-AROMA (Pruim et al., 2015a, 2015b) and found that the resulting subtypes still differed in mean FD, *t*(264) = −3.49, *p* < 0.001, and percentage of frames exceeding 0.2 mm, *t*(264) = −3.96, *p* < 0.001. Furthermore, regional artifacts were evident in the cluster centroids when the data were preprocessed without global signal regression (Supporting Information Figure S2A, Easson et al., 2019), and without global signal regression but with ICA denoizing using ICA-AROMA (Supporting Information Figure S2B, Easson et al., 2019).

The time series of 160 4.5-mm spherical regions of interest (ROIs) from the Dosenbach atlas (Dosenbach et al., 2010) were obtained (see Supporting Information Table S2 and Supporting Information Figure S1, Easson et al., 2019). Regions in this atlas were selected from meta-analyses of task-related fMRI studies and categorized into six different RSNs: the DMN, fronto-parietal network (FPN), CON, ON, SMN, and cerebellar network (CN). Additional details of the fMRI preprocessing steps can be found on the Preprocessed Connectomes Project website (http://www.preprocessed-connectomes-project.org/abide).

### Functional Connectivity

Each subject’s fMRI time series was truncated to 145 time points, which was the minimum number of time points across subjects. FC was defined by Fisher *z*-transformed Pearson correlations for each ROI pair across all time points for each participant. The effects of age and acquisition site (represented as a Helmert basis) were regressed out of the FC matrices. As it has been recently shown that despite implementing preprocessing steps that aim to correct for head motion in resting-state fMRI, residual motion effects can contaminate FC estimates (Ciric et al., 2017), a multivariate brain-behavior analysis was performed to determine if there were relationships between FC and head motion metrics (mean FD and percentage of frames exceeding 0.2 mm). There was not a significant relationship between FC and motion (*p* = 0.57).

### *K*-Means Clustering

*K*-means clustering was used to define subtypes of distinct FC patterns. The lower triangle of each participant’s FC matrix was used, such that the matrix for *k* means was in the form subjects × FC. The *k*-means algorithm begins with an initialization of *k* centroids. Then, in the *assignment* step, each participant is assigned to the closest centroid by using the cosine distance, defined as one minus the cosine of the included angle between each subject’s FC values and each cluster’s centroids, which are treated as vectors. Next, in the *centroid update* step, new centroids are defined as the mean of the data points that are currently assigned to that centroid. These two steps are repeated iteratively until convergence, when cluster assignments no longer change.

The “elbow point” criterion was used to determine the optimal number of clusters. To determine the elbow point, the average cosine distance between a cluster’s centroids and the FC values of participants assigned to that particular cluster is calculated for each cluster, then averaged across clusters to obtain a single distance metric for each value of *k*. These distances are then plotted as a function of *k*, and the “elbow” is defined as the value of *k* where the change in the rate of decrease in distance is sharpest. Values from *k* = 2 to *k* = 8 were tested (but also included *k* = 1 in the elbow point plot as a reference point). Furthermore, we evaluated the reliability of the number of clusters by using bootstrap resampling. Fifty percent of the sample was selected at random, and these were grouped into subtypes using the *k*-means algorithm for values of *k* from 2 to 8. The elbow criterion was then used to select the ideal value of *k* for the bootstrap sample. This process was repeated 500 times to determine the reliability of the optimal number of clusters.

### Partial Least Squares

Partial least squares (PLS) is a multivariate statistical technique that is used to optimally relate brain activity to experimental design or group membership in the form of latent variables (McIntosh et al., 1996; McIntosh & Lobaugh, 2004; Krishnan et al., 2011). PLS software, which is implemented in Matlab, is available for download from research.baycrest.org/pls-software. In *mean-centering PLS*, patterns relating a matrix of brain variables (in the form subjects × brain variables) and group membership are calculated. For this study, the brain variables were the FC values in the lower triangle of each subject’s FC matrix (12,720 connections). Mean-centering PLS was used to examine differences in FC between subtypes and between ASD and TD participants.

By using singular value decomposition (SVD), orthogonal patterns that express the maximal covariance between the brain variables and group membership are computed. The resulting patterns are sorted in order of the proportion of covariance between the brain and design/behavior variables that the pattern accounts for, with the first pattern accounting for the most covariance. Each pattern consists of saliences (weights) and a singular value. The brain saliences indicate which brain variables (in this case, functional connections) best characterize the relationship between the brain variables and group differences. Design saliences indicate the group differences profiles that best characterize this relationship. Singular values indicate the proportion of covariance between the brain and design matrices that each pattern accounts for. Brain scores, which represent each subject’s contribution to each pattern, are calculated by multiplying the original matrix of brain variables by the brain saliences.

In *behavior PLS*, a matrix of behavior variables is also included in the analysis to determine design-dependent (in this case, group-dependent) relationships between the brain variables and behavior. For this study, behavioral PLS was used to examine associations between FC and a set of behavioral variables including IQ, ADOS scores (communication, social affect, and RRBs), and scores on the SRS in each subtype.

The statistical significance of each pattern was determined using permutation testing. For this procedure, the rows (participants) of the matrix of brain variables are reshuffled, and new singular values are obtained using SVD. In this study, this procedure was repeated 1,000 times to create a distribution of singular values. The *p* value associated with the original singular value is defined as the proportion of singular values from the sampling distribution that are greater than the original singular value, thus representing the probability of obtaining a singular value larger than the original value under the null hypothesis that there is no association between the brain variables and group membership.

In addition to determining the statistical significance of each pattern, the reliability of the brain saliences can also be determined by utilizing a bootstrapping procedure. Bootstrap samples are generated by randomly sampling subjects with replacement, while ensuring that group membership is maintained.In this study, 500 bootstrap samples were generated.Creating bootstrap samples allows one to determine which brain variables are stable, regardless of which participants are included in the analysis. The BSR, defined as the ratio of the brain salience to the standard error of the salience (as estimated by the bootstrap procedure), is a measure of this stability. Reliable connections were defined as those that surpassed a BSR threshold of ±2.0, which corresponds roughly to a 95% confidence interval.

As FC values can take on positive or negative values, positive BSRs could correspond to either stronger positive or weaker negative connectivity in one group compared with the other, and negative BSRs could indicate weaker positive or stronger negative connectivity. Thus, expressions of FC PLS contrasts were generated for each group. Positive expressions were generated by averaging connections (Fisher *z*-transformed Pearson correlation coefficients) that had BSRs greater than 2 across all participants in each group. A similar procedure was performed for negative expressions, that is, for connections showing BSRs less than −2.

In addition to assessing the contribution of each individual connection to the group differences, we were interested in determining the extent to which network-level FC, both within and between RSNs, contributed to the group differences. This was of particular interest because of hypotheses that ASD may be characterized by atypical FC within and between networks (e.g., Hull et al., 2016; Rudie & Dapretto, 2013b). To assess the relative contributions of each RSN to the spatial patterns, the BSR-thresholded spatial maps (i.e., adjacency matrices in the form connections × connections) were separated into positive BSRs and negative BSRs. These maps were thresholded such that connections with a BSR less than 2 but greater than −2 were set to 0. Positive BSRs greater than 2 were set to 1, and negative BSRs less than −2 were set to − 1. All thresholded BSRs within each pair of networks were then averaged to obtain a 6 × 6 matrix showing the average contribution of each network pair to the spatial pattern, separately for positive and negative BSRs. To assess the significance of these contributions, the order of connections in the BSR-thresholded matrices was permuted while keeping the RSN labels the same, and then the above procedure was repeated to calculate the RSN contributions. This process was repeated 1,000 times to obtain a distribution of average contribution values for each RSN pair. Then, the significance of the original contribution is defined as the proportion of contribution values from the sampling distribution that are greater than or equal to the original value.

### Data Visualization

Connectivity circle plots were created using the plot_connectivity_circle function from the open-source MNE software package implemented in Python (Gramfort et al., 2013; 2014). All other figures were created using Matlab (MATLAB 8.6.0 [R2015b], MathWorks, Natick, MA). Violin plots were created using the distributionPlot.m function (Jonas, 2017).

## ACKNOWLEDGMENTS

The authors thank Bratislav Misic and Sam Doesburg for helpful discussions, and the contributors to the Autism Brain Imaging Exchange and Preprocessed Connectomes Project.

## AUTHOR CONTRIBUTIONS

Amanda K. Easson: Conceptualization; Formal analysis; Methodology; Visualization; Writing – original draft; Writing – review & editing. Zainab Fatima: Conceptualization; Methodology; Writing – review & editing.Anthony R. McIntosh: Conceptualization; Methodology; Supervision; Writing – review & editing.

## FUNDING INFORMATION

Anthony R. McIntosh, Natural Sciences and Engineering Research Council of Canada (http://dx.doi.org/10.13039/501100000038), Award ID: RGPIN-2018-04457. Amanda K. Easson, Ontario Graduate Scholarship. Amanda K. Easson, Mynne & Harold Soupcoff Fellowship. Amanda K. Easson, Finkler Graduate Student Fellowship.

## TECHNICAL TERMS

Functional connectivity: The statistical association between the time series of activity of two brain regions.
Resting-state network: A set of brain regions that exhibit synchronous patterns of activity and are involved in similar functions.
*K*-means clustering: A machine learning method used to identify distinct patterns (clusters) in a set of data.
Bootstrapping: A resampling method in which statistics are recalculated on a random sample of data while maintaining group membership.
Permutation testing: A resampling method in which statistics are recalculated on data with shuffled group labels to obtain a null distribution to which the original test statistic is compared.
Partial least squares (PLS): A statistical method for characterizing relationships between a set of brain variables and group membership or a set of behaviors.
Latent variables: “Hidden” variable within observable variables that are not measured directly, but rather are inferred via statistical procedures.
