# Supplementary material for: Functional connectivity-based subtypes of individuals with and without autism spectrum disorder
Source: Netw Neurosci. 2019 Feb 1;3(2):344–62. doi: 10.1162/netn_a_00067 (PMC6370474; doi:10.1162/netn_a_00067)
Supplement: Supplementary file 1 [file netn-03-344-s001.pdf]

Easson, A. K., Fatima, Z., & McIntosha, A. R. (2019). Supporting Information for “Functional connectivity-based subtypes of individuals with and without autism spectrum disorder.” *Network Neuroscience*. *3*(2), 344–362. https://doi.org/10.1162/netn_a_00067

SUPPLEMENTARY MATERIALS

Supplementary Table 1: Participant characteristics by site

	NYU		SDSU		TRINITY		UM		USM	
**Variable**	**ASD Mean ± SD [range]**	**TD Mean ± SD [range]**	**ASD Mean ± SD [range]**	**TD Mean ± SD [range]**	**ASD Mean ± SD [range]**	**TD Mean ± SD [range]**	**ASD Mean ± SD [range]**	**TD Mean ± SD [range]**	**ASD Mean ± SD [range]**	**TD Mean ± SD [range]**
N	59	52	11	10	18	16	26	29	31	14
Age	14.49 ± 6.80 [7.13 – 39.10]	15.79 ± 6.66 [6.47 – 31.78]	15.00 ± 1.74 [12.13 – 17.15]	14.48 ± 1.31 [12.58 – 16.57]	16.69 ± 2.54 [13.58 – 21.42]	16.88 ± 4.15 [12.04 – 25.66]	13.92 ± 2.31 [9.20 – 18.60]	14.11 ± 3.75 [8.20 – 26.80]	22.75 ± 6.85 [11.35 – 35.71]	21.04 ± 6.06 [12.94 – 31.28]
IQ	108.47 ± 15.35 [80 – 148]	110.62 ± 11.87 [80 – 132]	116.36 ± 16.82 [81 – 141]	109.00 ± 9.85 [93 – 126]	110.50 ± 14.41 [89 – 135]	107.88 ± 13.88 [89 – 133]	109.44 ± 17.97 [77 – 147.5]	110.29 ± 8.06 [93.5 – 129]	99.45 ± 15.36 [76 – 132]	110.93 ± 14.25 [89 – 130]
Mean FD	0.07 ± 0.03 [0.03 – 0.14]	0.06 ± 0.03 [0.04 – 0.19]	0.06 ± 0.04 [0.02 – 0.14]	0.06 ± 0.02 [0.04 – 0.09]	0.09 ± 0.02 [0.07 – 0.14]	0.08 ± 0.02 [0.06 – 0.11]	0.07 ± 0.03 [0.02 – 0.16]	0.07 ± 0.03 [0.04 – 0.14]	0.09 ± 0.05 [0.02 – 0.19]	0.10 ± 0.03 [0.06 – 0.15]
Percent FD > 0.2mm	4.17 ± 4.70 [0 – 18.00]	3.26 ± 3.93 [0 – 18.00]	4.24 ± 6.16 [0 – 19.33]	1.80 ± 1.44 [0 – 4.67]	4.33 ± 3.42 [0 – 10.67]	3.71 ± 4.29 [0 – 16.67]	3.92 ± 4.67 [0 – 18]	4.32 ± 4.44 [0 – 16]	6.69 ± 6.91 [0 – 18.67]	7.29 ± 5.09 [0 – 19.33]
Handedness	44 RH 14 LH	48 RH 4 LH	10 RH 1 LH	8 RH 2 LH	18 RH 0 LH	16 RH 0 LH	26 RH 0 LH	29 RH 0 LH	29 RH 2 LH	14 RH 0 LH
Eye status	53 open 6 closed	42 open 10 closed	11 open 0 closed	10 open 0 closed	0 open 18 closed	0 open 16 closed	19 open 4 closed	23 open 4 closed	31 open 0 closed	14 open 0 closed
Medication use	14 yes 45 no 0 unknown	0 yes 52 no 0 unknown	1 yes 10 no 0 unknown	0 yes 10 no 0 unknown	0 yes 18 no 0 unknown	0 yes 16 no 0 unknown	12 yes 13 no 1 unknown	0 yes 28 no 1 unknown	0 yes 0 no 31 unknown	0 yes 0 no 14 unknown
Comorbidities	28 yes 31 no/unknown	0 yes 52 no/unknown	0 yes 11 no/unknown	0 yes 10 no/unknown	0 yes 18 no/unknown	0 yes 16 no/unknown	0 yes 26 no/unknown	0 yes 29 no/unknown	0 yes 31 no/unknown	0 yes 14 no/unknown

ADOS Total	11.56 ± 4.19<br>[5 – 22]<br>(N = 59)	N/A	11.20 ± 3.94<br>[7 – 19]<br>(N = 10)	N/A	11.22 ± 2.86<br>[7 – 17]<br>(N = 18)	N/A	N/A	N/A	12.39 ± 2.64<br>[6 – 18]<br>(N = 31)	1.14 ± 1.17<br>[0 – 4]<br>(N = 14)
ADOS Communication	3.71 ± 1.57<br>[0 – 8]<br>(N = 59)	N/A	3.60 ± 2.07<br>[0 – 7]<br>(N = 10)	N/A	N/A	N/A	N/A	N/A	4.32 ± 1.28<br>[2 – 7]<br>(N = 31)	0.50 ± 0.64<br>[0 – 3]<br>(N = 14)
ADOS Social	7.85 ± 3.03<br>[2 – 14]<br>(N = 59)	N/A	7.60 ± 3.34<br>[3 – 13]<br>(N = 10)	N/A	N/A	N/A	N/A	N/A	8.06 ± 2.22<br>[4 – 12]<br>(N = 31)	0.64 ± 0.84<br>[0 – 2]<br>(N = 14)
ADOS RRB	2.29 ± 1.52<br>[0 – 7]<br>(N = 59)	N/A	2.00 ± 1.15<br>[0 –4]<br>(N = 10)	N/A	N/A	N/A	N/A	N/A	1.55 ± 1.35<br>[0 – 5]<br>(N = 29)	0.07 ± 0.27<br>[0 – 1]<br>(N = 14)
ADI-R Social	18.96 ± 5.76<br>[7 – 28]<br>(N = 55)	N/A	17.89 ± 4.57<br>[12 – 23]<br>(N = 9)	N/A	20.06 ± 5.87<br>[10 – 30]<br>(N = 18)	N/A	19.04 ± 4.85<br>[9 – 26]<br>(N = 26)	N/A	N/A	N/A
ADI-R Verbal	15.39 ± 4.36<br>[8 – 25]<br>(N = 56)	N/A	13.11 ± 5.71<br>[2 – 20]<br>(N = 9)	N/A	15.44 ± 4.88<br>[8 – 23]<br>(N = 18)	N/A	16.08 ± 3.38<br>[9 – 22]<br>(N = 26)	N/A	N/A	N/A
ADI-R RRB	5.46 ± 2.66<br>[0 – 12]<br>(N = 56)	N/A	6.11 ± 1.76<br>[3 – 9]<br>(N = 9)	N/A	5.28 ± 2.40<br>[2 – 11]<br>(N = 18)	N/A	6.19 ± 2.86<br>[1 – 12]<br>(N = 26)	N/A	N/A	N/A
SRS	91.40 ± 30.51<br>[34 – 164]<br>(N = 58)	23.80 ± 12.36<br>[5 – 56]<br>(N = 35)	N/A	N/A	N/A	N/A	N/A	N/A	94.74 ± 32.30<br>[26 – 148]<br>(N = 31)	12.57 ± 8.57<br>[1– 31]<br>(N = 14)

Supplementary Table 2: Dosenbach Atlas (from Dosenbach et al., 2010)

ROI #	Label	MNI Coordinates (x, y, z)			RSN
1	aPFC	-25	51	27	DMN
2	mPFC	0	51	32	DMN
3	vmPFC	6	64	3	DMN
4	vmPFC	9	51	16	DMN
5	vmPFC	-6	50	-1	DMN
6	vmPFC	-11	45	17	DMN
7	vmPFC	8	42	-5	DMN
8	vlPFC	46	39	-15	DMN
9	sup frontal	23	33	47	DMN
10	sup frontal	-16	29	54	DMN
11	ACC	9	39	20	DMN
12	post cingulate	1	-26	31	DMN
13	post cingulate	-8	-41	3	DMN
14	post cingulate	-5	-43	25	DMN
15	post cingulate	-5	-52	17	DMN
16	post cingulate	10	-55	17	DMN
17	post cingulate	-11	-58	17	DMN
18	precuneus	-3	-38	45	DMN
19	precuneus	9	-43	25	DMN
20	precuneus	5	-50	33	DMN
21	precuneus	-6	-56	29	DMN
22	precuneus	11	-68	42	DMN
23	IPS	-36	-69	40	DMN
24	angular gyrus	51	-59	34	DMN
25	angular gyrus	-48	-63	35	DMN
26	fusiform	28	-37	-15	DMN
27	inf temporal	52	-15	-13	DMN
28	inf temporal	-59	-25	-15	DMN
29	inf temporal	-61	-41	-2	DMN
30	occipital	-28	-42	-11	DMN
31	occipital	-9	-72	41	DMN
32	occipital	45	-72	29	DMN
33	occipital	-2	-75	32	DMN
34	occipital	-42	-76	26	DMN
35	aPFC	29	57	18	FPN

36	aPFC	-29	57	10	FPN
37	vent aPFC	42	48	-3	FPN
38	vent aPFC	-43	47	2	FPN
39	vlPFC	39	42	16	FPN
40	dlPFC	40	36	29	FPN
41	dlPFC	46	28	31	FPN
42	dlPFC	-44	27	33	FPN
43	vPFC	-52	28	17	FPN
44	ACC	-1	28	40	FPN
45	dFC	40	17	40	FPN
46	dFC	44	8	34	FPN
47	dFC	-42	7	36	FPN
48	IPL	-41	-40	42	FPN
49	IPL	54	-44	43	FPN
50	IPL	-48	-47	49	FPN
51	IPL	-53	-50	39	FPN
52	IPL	44	-52	47	FPN
53	post parietal	-35	-46	48	FPN
54	IPS	-32	-58	46	FPN
55	IPS	32	-59	41	FPN
56	aPFC	27	49	26	CON
57	vPFC	34	32	7	CON
58	vFC	51	23	8	CON
59	vFC	-46	10	14	CON
60	vFC	-48	6	1	CON
61	mFC	0	15	45	CON
62	dACC	9	20	34	CON
63	ACC	-2	30	27	CON
64	post cingulate	-4	-31	-4	CON
65	ant insula	38	21	-1	CON
66	ant insula	-36	18	2	CON
67	mid insula	37	-2	-3	CON
68	mid insula	32	-12	2	CON
69	mid insula	-30	-14	1	CON
70	post insula	-30	-28	9	CON
71	basal ganglia	-6	17	34	CON
72	basal ganglia	-20	6	7	CON

73	basal ganglia	14	6	7	CON
74	basal ganglia	11	-24	2	CON
75	thalamus	-12	-3	13	CON
76	thalamus	-12	-12	6	CON
77	thalamus	11	-12	6	CON
78	precuneus	8	-40	50	CON
79	angular gyrus	-41	-47	29	CON
80	fusiform	54	-31	-18	CON
81	parietal	58	-41	20	CON
82	parietal	-55	-44	30	CON
83	TPJ	-52	-63	15	CON
84	sup temporal	42	-46	21	CON
85	temporal	51	-30	5	CON
86	temporal	43	-43	8	CON
87	temporal	-59	-47	11	CON
88	temporal	46	-62	5	ON
89	occipital	-18	-50	1	ON
90	occipital	-34	-60	-5	ON
91	occipital	36	-60	-8	ON
92	occipital	-44	-63	-7	ON
93	occipital	19	-66	-1	ON
94	occipital	17	-68	20	ON
95	occipital	39	-71	13	ON
96	occipital	29	-73	29	ON
97	occipital	-29	-75	28	ON
98	occipital	-16	-76	33	ON
99	occipital	9	-76	14	ON
100	occipital	15	-77	32	ON
101	occipital	20	-78	-2	ON
102	post occipital	-5	-80	9	ON
103	post occipital	29	-81	14	ON
104	post occipital	33	-81	-2	ON
105	post occipital	-37	-83	-2	ON
106	post occipital	-29	-88	8	ON
107	post occipital	13	-91	2	ON
108	post occipital	27	-91	2	ON
109	post occipital	-4	-94	12	ON

110	frontal	58	11	14	SMN
111	frontal	53	-3	32	SMN
112	dFC	60	8	34	SMN
113	vFC	-55	7	23	SMN
114	vFC	43	1	12	SMN
115	pre-SMA	10	5	51	SMN
116	SMA	0	-1	52	SMN
117	precentral gyrus	58	-3	17	SMN
118	precentral gyrus	-44	-6	49	SMN
119	precentral gyrus	46	-8	24	SMN
120	precentral gyrus	-54	-9	23	SMN
121	precentral gyrus	44	-11	38	SMN
122	precentral gyrus	-54	-22	22	SMN
123	mid insula	-42	-3	11	SMN
124	mid insula	33	-12	16	SMN
125	mid insula	-36	-12	15	SMN
126	post insula	42	-24	17	SMN
127	parietal	-26	-8	54	SMN
128	parietal	-47	-12	36	SMN
129	parietal	-38	-15	59	SMN
130	parietal	-47	-18	50	SMN
131	parietal	46	-20	45	SMN
132	parietal	-55	-22	38	SMN
133	parietal	41	-23	55	SMN
134	parietal	18	-27	62	SMN
135	parietal	-38	-27	60	SMN
136	parietal	-24	-30	64	SMN
137	post parietal	-41	-31	48	SMN
138	sup parietal	34	-39	65	SMN
139	temporal	59	-13	8	SMN
140	temporal	-54	-22	9	SMN
141	temporal	-41	-37	16	SMN
142	temporal	-53	-37	13	SMN
143	lat cerebellum	-28	-44	-25	CN
144	lat cerebellum	-24	-54	-21	CN
145	lat cerebellum	-34	-57	-24	CN
146	lat cerebellum	21	-64	-22	CN

147	med cerebellum	-6	-60	-15	CN
148	med cerebellum	-16	-64	-21	CN
149	med cerebellum	1	-66	-24	CN
150	med cerebellum	-11	-72	-14	CN
151	med cerebellum	5	-75	-11	CN
152	med cerebellum	14	-75	-21	CN
153	inf cerebellum	-37	-54	-37	CN
154	inf cerebellum	-25	-60	-34	CN
155	inf cerebellum	32	-61	-31	CN
156	inf cerebellum	-34	-67	-29	CN
157	inf cerebellum	33	-73	-30	CN
158	inf cerebellum	-21	-79	-33	CN
159	inf cerebellum	-6	-79	-33	CN
160	inf cerebellum	18	-81	-33	CN

Supplementary Table 3: Demographic and behaviour variables in FC-based subtypes

**Variable**	**Subtype 1 Mean ± SD [range]**	**Subtype 2 Mean ± SD [range]**	**Significance**
Age	16.55 ± 6.47 [6.47 – 35.71]	15.96 ± 5.46 [7.19 – 39.10]	$t(264) = 0.79$, $p = 0.43$
IQ	107.62 ± 14.79 [80 – 148]	109.91 ± 13.68 [76 – 142]	$t(264) = -1.31$ $p = 0.19$
Mean FD	0.07 ± 0.03 [0.02 – 0.19]	0.07 ± 0.04 [0.03 – 0.19]	$t(264) = 0.37$, $p = 0.71$
Percent FD ≥ 0.2mm	4.71 ± 5.10 [0 – 19.33]	3.93 ± 4.55 [0 – 19.33]	$t(264) = 1.31$, $p = 0.19$
Handedness	120 RH<br>21 LH	109 RH<br>10 LH	$X^2(1, N=266) = 0.52$, $p = 0.13$

Eye status	109 open<br>30 closed	107 open<br>20 closed	$X^2(1, N=266) = 0.68, p = 0.27$
Scan Site	NYU: 61<br>SDSU: 11<br>TRINITY: 17<br>UM: 26<br>USM: 24	NYU: 50<br>SDSU: 10<br>TRINITY: 17<br>UM: 29<br>USM: 21	$X^2(4, N=266) = 0.96, p = 0.92$
Medication use	14 yes<br>100 no/unknown	13 yes<br>92 no/unknown	$X^2(1, N=266) = 0.99, p \sim 1$
Comorbidities	16 yes<br>123 no/unknown	12 yes<br>115 no/unknown	$X^2(1, N=266) = 1.25, p = 0.69$
ADOS Total (ASD and TD)	11.30 $\pm$ 4.03<br>[1 – 22]<br>(N = 70)	9.76 $\pm$ 5.42<br>[0 – 19]<br>(N = 62)	$t(130) = 1.87, p = 0.06$
ADOS Communication (ASD and TD)	3.89 $\pm$ 1.61<br>[0 – 8]<br>(N = 61)	3.00 $\pm$ 1.99<br>[0 – 7]<br>(N = 53)	$t(112) = 2.62, p = 0.01$
ADOS Social (ASD and TD)	7.34 $\pm$ 3.14<br>[0 – 14]<br>(N = 61)	6.60 $\pm$ 3.99<br>[0 – 13]<br>(N = 53)	$t(112) = 1.11, p = 0.27$
ADOS RRB (ASD and TD)	1.85 $\pm$ 1.51<br>[0 – 7]<br>(N = 59)	1.74 $\pm$ 1.55<br>[0 – 7]<br>(N = 53)	$t(110) = 0.39, p = 0.70$
ADOS Total (ASD)	11.59 $\pm$ 3.71<br>[5 – 22]<br>(N = 68)	11.84 $\pm$ 3.66<br>[5 – 19]<br>(N = 50)	$t(116) = -0.37, p = 0.71$
ADOS Communication (ASD)	3.98 $\pm$ 1.54<br>[0 – 8]	3.76 $\pm$ 1.58<br>[0 – 7]	$t(98) = 0.72, p = 0.47$

	(N = 59)	(N = 41)	
ADOS Social (ASD)	7.58 ± 2.92<br>[2 – 14]<br>(N = 59)	8.34 ± 2.61<br>[2 – 13]<br>(N = 41)	$t(98) = 1.34$, $p = 0.18$
ADOS RRB (ASD)	1.91 ± 1.49<br>[0 – 7]<br>(N = 57)	2.22 ± 1.42<br>[0 – 7]<br>(N = 41)	$t(96) = -1.03$, $p = 0.31$
ADI-R Social (ASD)	19.02 ± 5.46<br>[9 – 30]<br>(N = 62)	19.15 ± 5.46<br>[7 – 27]<br>(N = 46)	$t(106) = -0.13$, $p = 0.90$
ADI-R Verbal (ASD)	15.19 ± 4.77<br>[2 – 25]<br>(N = 62)	15.62 ± 3.81<br>[8 – 23]<br>(N = 47)	$t(107) = -0.50$, $p = 0.62$
ADI-R RRB (ASD)	5.65 ± 2.69<br>[0 – 12]<br>(N = 62)	5.68 ± 2.49<br>[1 – 12]<br>(N = 47)	$t(107) = -0.07$, $p = 0.94$
SRS (ASD and TD)	72.97 ± 44.59<br>[5 – 164]<br>(N = 72)	60.50 ± 49.58<br>[1 – 148]<br>(N = 66)	$t(136) = 1.71$, $p = 0.09$
SRS (ASD)	72.97 ± 44.59<br>[26 – 164]<br>(N = 51)	60.50 ± 40.98<br>[29 – 148]<br>(N = 38)	$t(87) = 0.64$, $p = 0.52$
SRS (TD)	20.95 ± 11.87<br>[5 – 46]<br>(N = 21)	20.32 ± 13.04<br>[1 – 56]<br>(N = 28)	$t(47) = 0.17$, $p = 0.86$

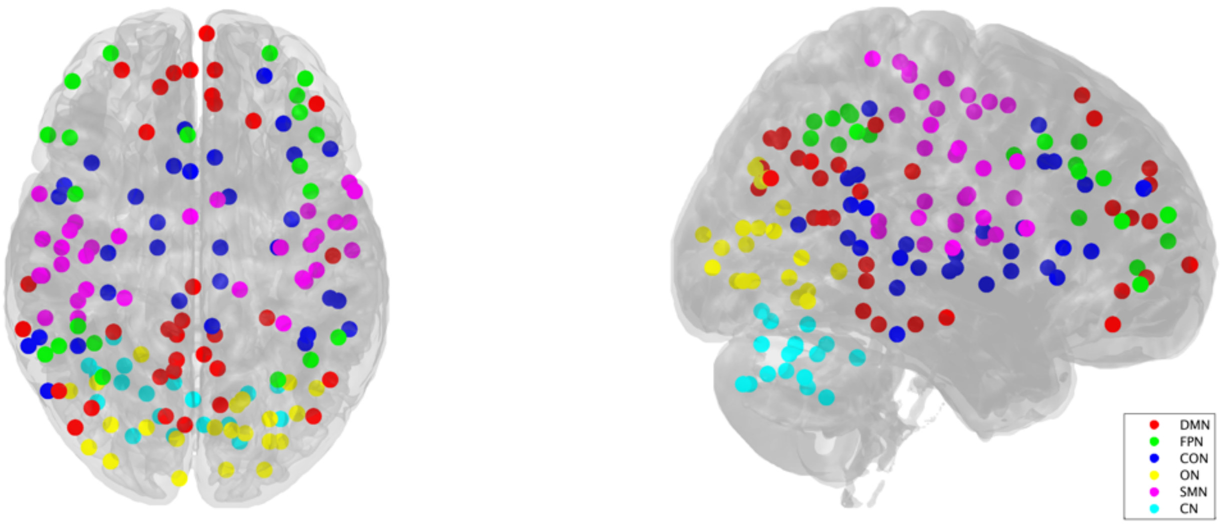

Supplementary Figure 1. Dosenbach ROIs.

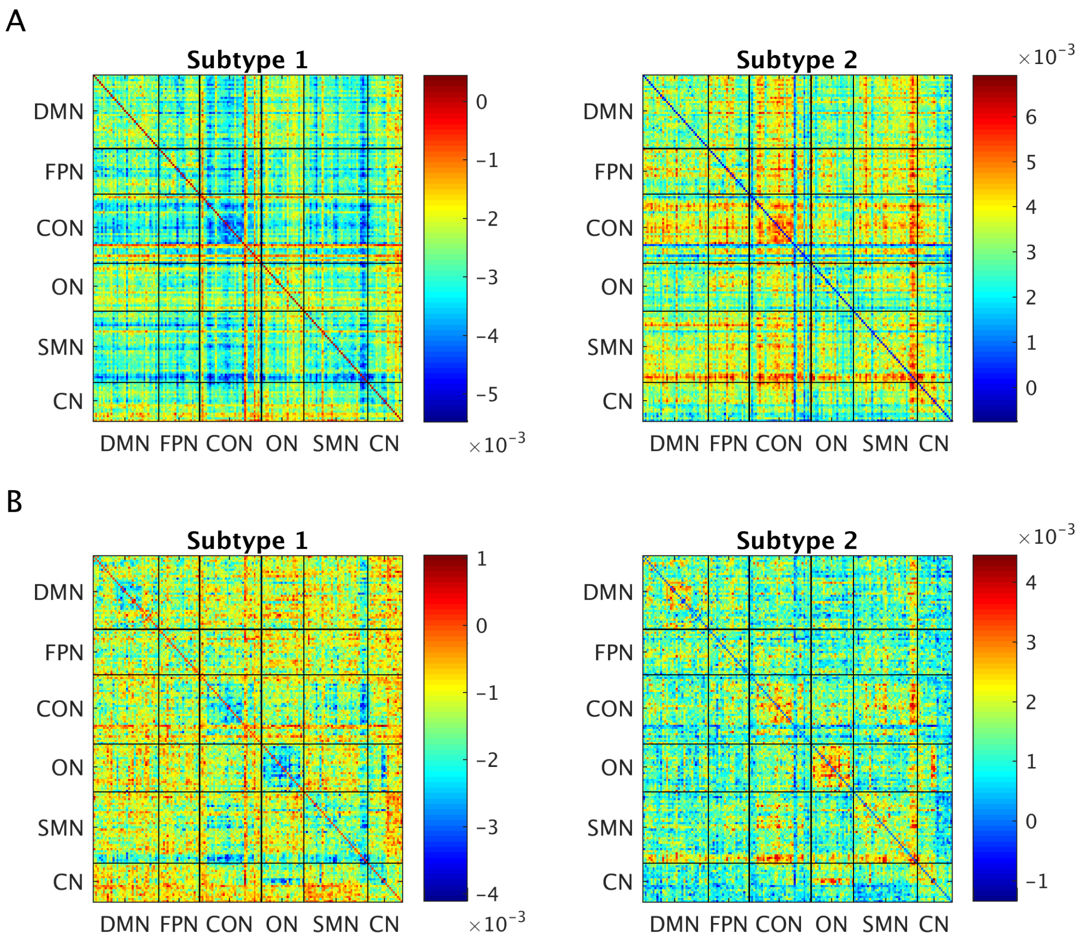

Supplementary Figure 2. Centroids using A) no global signal regression, B) no global signal regression + ICA AROMA.

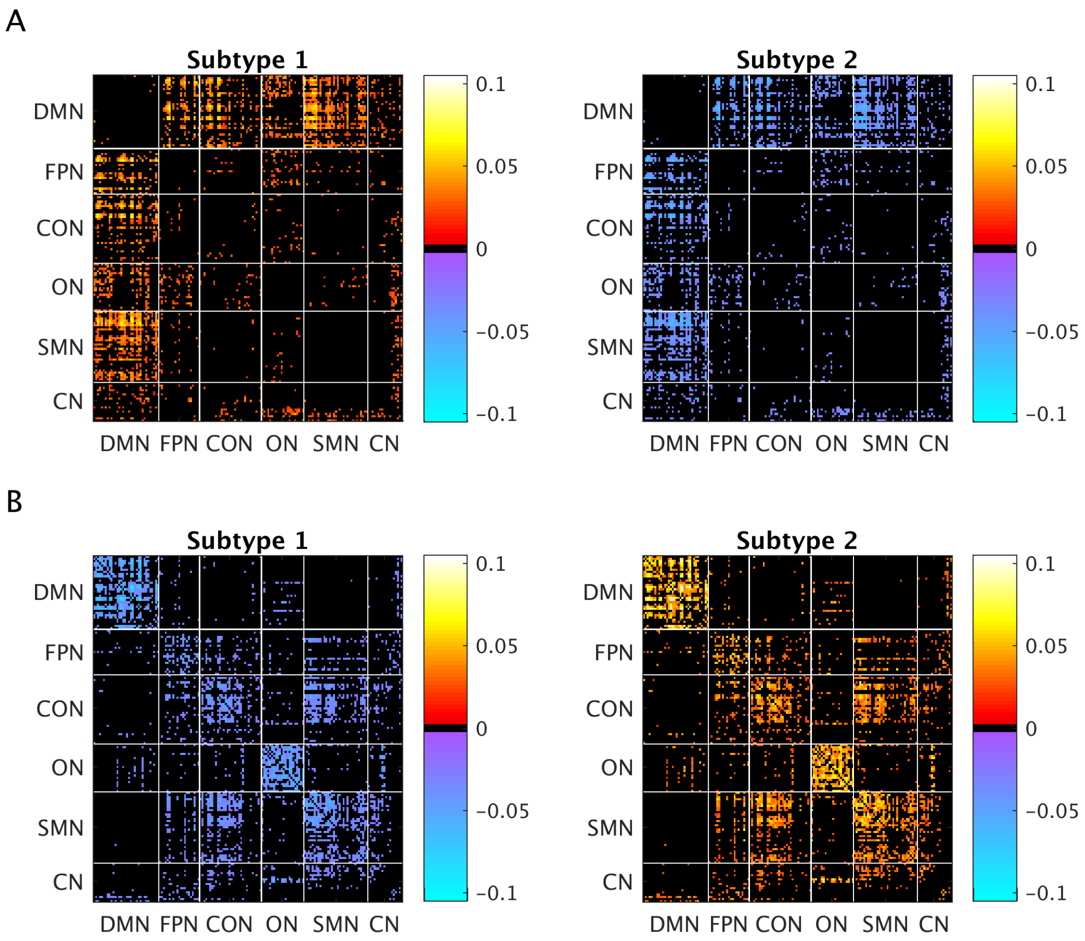

Supplementary Figure 3. FC contrast expressions for the first pattern from the multivariate group analysis, showing average Fisher z-transformed Pearson correlation coefficients for each subtype. A) Positive expression of the contrast (BSRs > 2). B) Negative expression of the contrast (BSRs < -2).

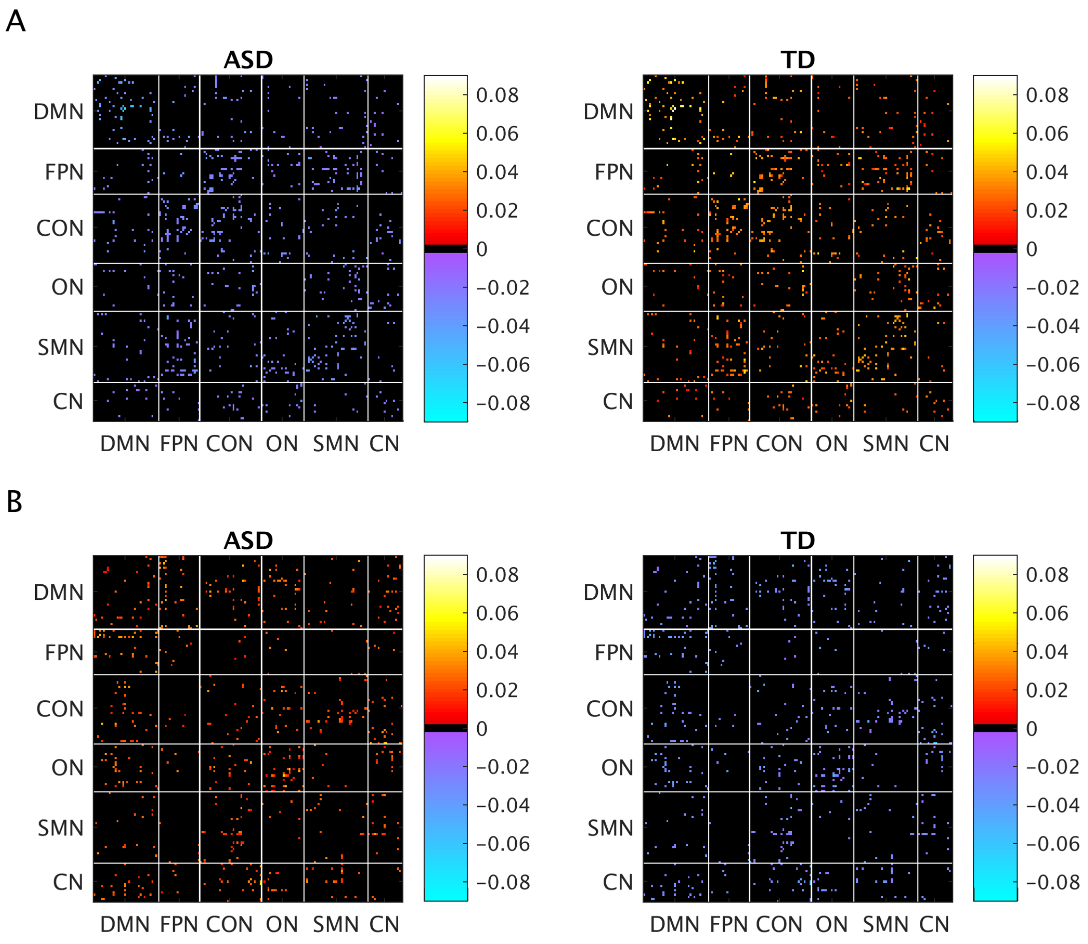

Supplementary Figure 4. FC contrast expressions for the second pattern from the multivariate group analysis. A) Positive expression of the contrast (BSRs > 2). B) Negative expression of the contrast (BSRs < -2).

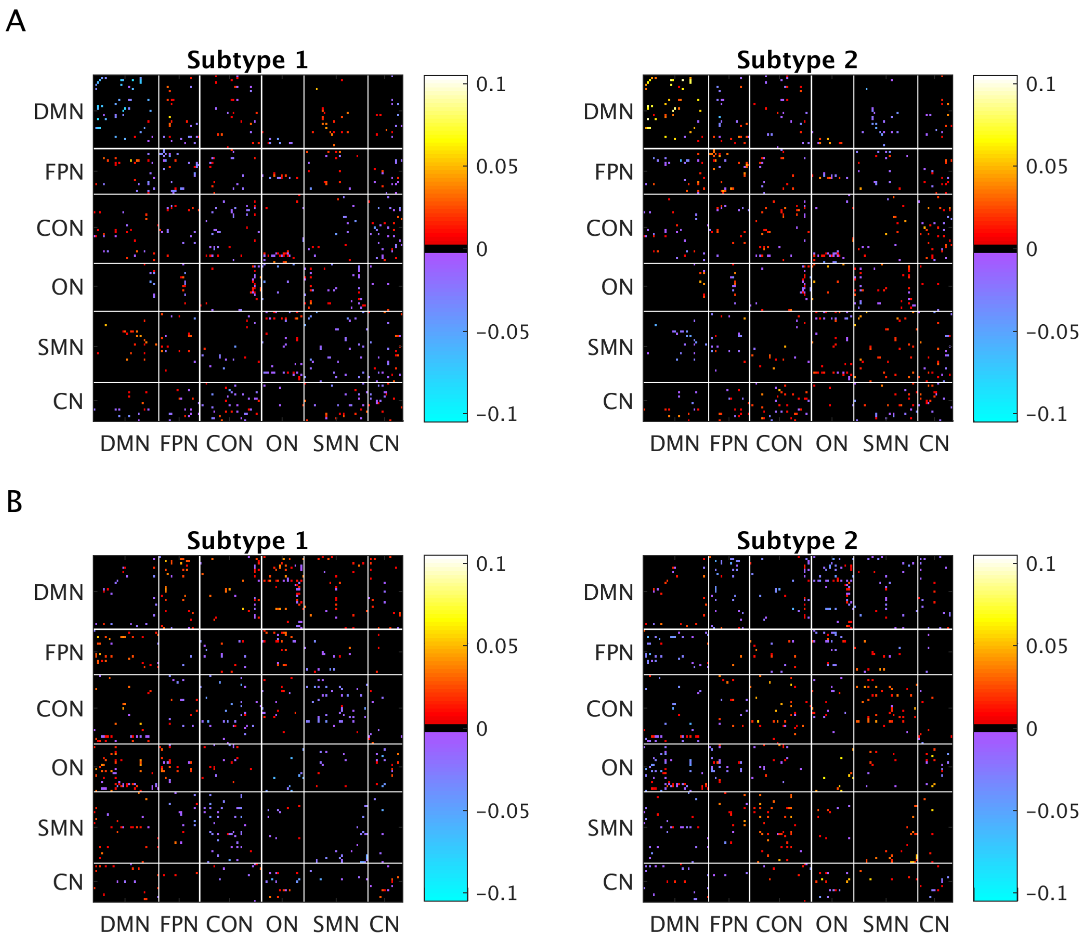

Supplementary Figure 5. FC contrast expressions for first pattern from the multivariate brain-behaviour analysis. A) Positive expression of the contrast (BSRs > 2). B) Negative expression of the contrast (BSRs < -2).

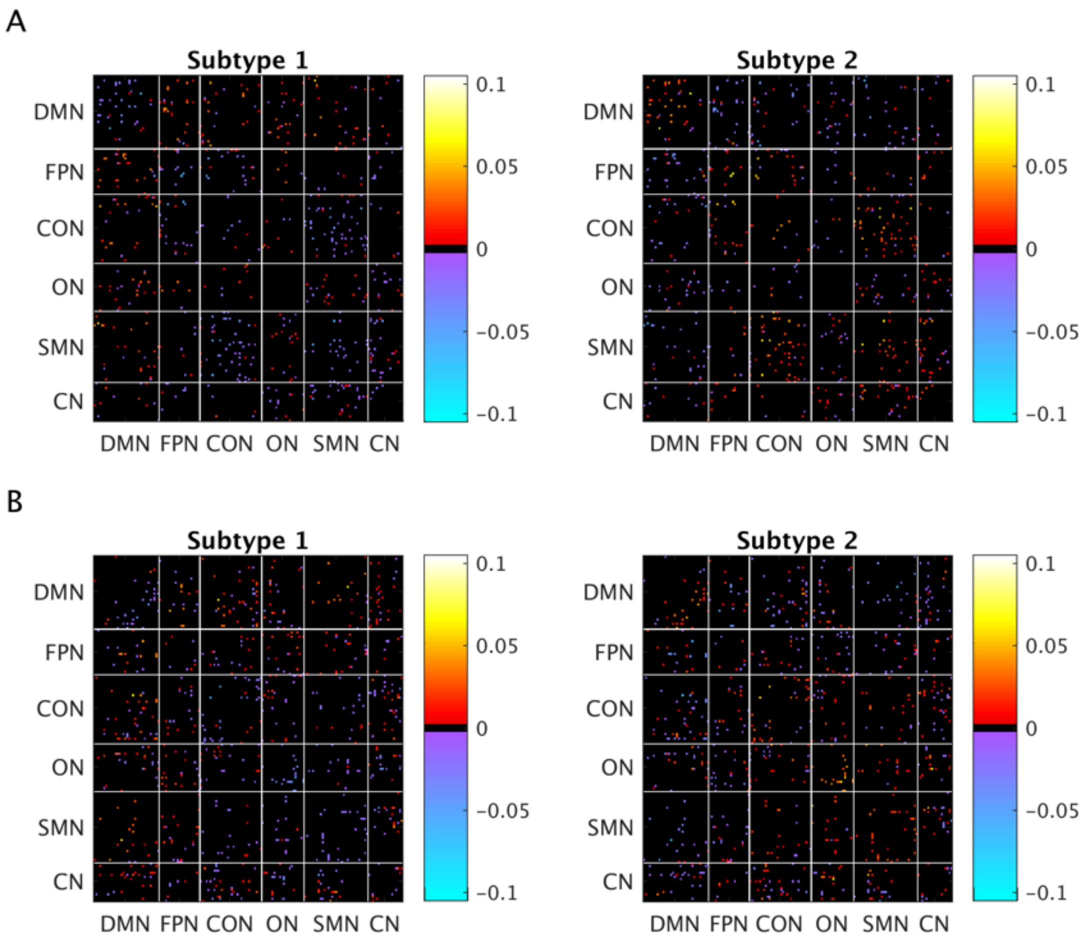

Supplementary Figure 6. FC contrast expressions for the third pattern from the multivariate brain-behaviour analysis. A) Positive expression of the contrast (BSRs > 2). B) Negative expression of the contrast (BSRs < -2).
